# Supplementary material for: In Vivo Treatment with Insulin-like Growth Factor 1 Reduces CCR5 Expression on Vaccine-Induced Activated CD4+ T-Cells
Source: Vaccines (Basel). 2023 Oct 30;11(11):1662. doi: 10.3390/vaccines11111662 (PMC10675829; doi:10.3390/vaccines11111662)
Supplement: Supplementary file 1 [file vaccines-11-01662-s001.zip › vaccines-2615028-supplementary.pdf]

In vivo treatment with insulin-like growth factor reduces CCR5 expression on vaccine-induced activated CD4$^+$ T-cells

Massimiliano Bissa [1,*], Veronica Galli [1], Luca Schifanella [1], Monica Vaccari [1,2], Mohammad Arif Rahman [1], Giacomo Gorini [1], Nicolo Binello [1], Sarkis Sarkis [1], Anna Gutowska [1], Isabela Silva de Castro [1], Melvin N. Doster [1], Ramona Moles [1], Guido Ferrari [3], Xiaoying Shen [3], David C. Montefiori [3], Kombo F. N'guessan [4,5], Dominic Paquin-Proulx [4,5], Pamela A. Kozlowski [8], David J. Venzon [7], Hyoyoung Choo-Wosoba [7], Matthew W. Breed [8], Joshua Kramer [8], Genoveffa Franchini [1,*]

Supplementary Material

Figure S1. IGF-1 effect on antibody responses to gp120 proteins and V1/V2 scaffolds. (**a-c**) Specific activity to gp70 SIV V1/V2 of (**a**) mac239cs, 23, (**b**) mac251WY30, and (**c**) smE660-BR-CG7V in rectal secretions of vaccinated (n=13) and vaccinated+IGF-1 (n=12) animals at week 14. (**d**) Specific activity to gp70 SIV V1/V2 of smE660-BR-CG7V in vaginal secretions of vaccinated (n=13) and vaccinated+IGF-1 (n=12) animals at week 14. Comparisons: two-tailed Mann-Whitney U test with mean and SD. Statistical significance: *P<0.05; **P<0.01; ***P<0.001; ****P<0.0001.

Figure S2. IGF-1 effect on ADCC and NK function. (**a, b**) Normalized ADCC killing in plasma of vaccinated (n=13) and vaccinated+IGF-1 (n=12) animals at week 14 against target cells coated with (**a**) SIV_{mac251} V1 deleted gp120 or (**b**) wild-type proteins. (**c**) Frequencies of non-classical monocytes in blood of vaccinated (n=13) and vaccinated+IGF-1 (n=12) animals at week 13. (**d, e**) Frequencies of NKG2A$^+$ NK cells expressing Granzyme B, Perforin, INF-γ or TNF-α in (**d**) unstimulated or (**e**) PMA+I stimulated PBMCs, with or without IGF-1, collected from vaccinated (n=8) animals following last immunization (week 14). Comparisons: two-tailed Wilcoxon signed rank test between gp120 and gp120+IGF-1 treated cells for secreted protein (unadjusted p values) with mean and SD. (**f**) Correlation between the Specific ADCC killing of SIV infected cells in plasma of vaccinated animals (n=13) at week 14 and the trogocytosis score in plasma at week 14. (**g**) Frequencies of CD14$^+$ efferocytes following *in vitro* 24 hours incubation without (NS) or with IGF-1 stimulation (IGF-1) in CD14$^+$ cells isolated from naïve macaques (n=9). Comparisons: (**a-c**) two-tailed Mann-Whitney U test with mean and SD; (**g**) two-tailed Wilcoxon signed rank test with mean and SD; (**f**) two-tailed Spearman correlation with simple linear regression. Statistical significance: *P<0.05; **P<0.01; ***P<0.001; ****P<0.0001.

Table S1. Plasma IgA, IgG1, IgG2, IgG3, IgG4, and the sum of IgGs response to SIV_{mac251} gp120. The data represented show the median, the 25th and 75th percentiles, and the 95% Confidence Intervals (CI) of the µg/ml of IgA and IgGs measured in plasma of vaccinated (n=13) and vaccinated+IGF-1 (n=12) animals at week 14. The last column shows the unadjusted p-values of the comparisons between the two groups using two-tailed Mann-Whitney U test.

Table S2. Serum neutralizing antibody responses to SIV viruses. The data represented show the median, the 25th and 75th percentiles, and the 95% Confidence Intervals (CI) of ID_{50} and ID_{80} neutralizing antibody responses to different SIV viruses (tier 1A/SIV_{mac251}, tier 1A/SIV_{smE660}, tier 1B/SIV_{smE660} and SIV_{mac251} used for the animal challenge) in serum of vaccinated (n=13) and

vaccinated+IGF-1 (n=12) animals at week 14. The last column shows the unadjusted p-values of the comparisons between the two groups using two-tailed Mann-Whitney U test. Rows highlighted in red or blue indicate a higher response in vaccine or vaccine+IGF-1 animals, respectively.

Table S3. Variable regions 1 and 2 overlapping peptides. Amino acid sequence (single letter aa code) of 15 overlapping peptides (20-mers) encompassing the V1 (peptides 15-24) and V2 (peptides 25-29) of $SIV_{M766-mac251}$.

Table S4. Binding Antibody Multiplex assay (BAMA) in vaccinated and vaccinated+IGF-1 animals. The data represented show the median, the 25th and 75th percentiles, the 95% confidence intervals (CI) of each antibody response to SIV proteins (p55, gp41, gp120 of SIV_{mac239}, gp140 of SIV_{smE660}, and gp130 of SIV_{mac251}) and SIV gp70-V1/V2 scaffolds ($SIV_{mac239cs,}$ 23, $SIV_{mac251WY30}$ and $SIV_{smE660-BR-CG7V}$), measured by BAMA in plasma, rectal and vaginal secretions of vaccinated and vaccinated+IGF-1 animals at 2 weeks following the last immunization (week 14). The N of animals used in each analysis is reported. The last column shows the unadjusted p-values of the comparisons between the two groups using the two-tailed Mann-Whitney U test. Rows highlighted in blue indicate higher response in vaccinated+IGF-1 animals.

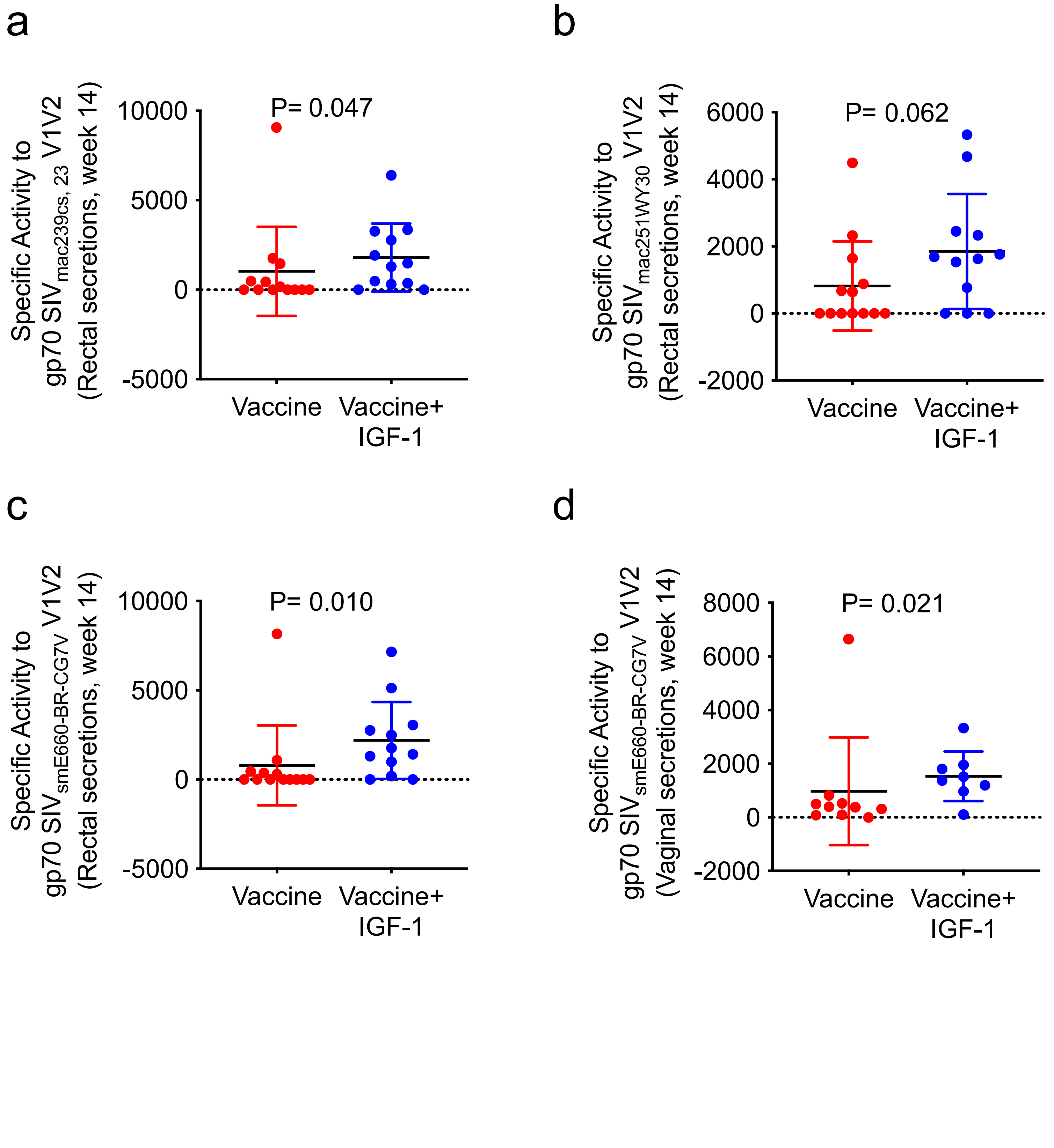
a
Specific Activity to gp70 SIVmac239cs, 23 V1V2 (Rectal secretions, week 14)
P= 0.047
10000
5000
0
-5000
Vaccine
Vaccine+ IGF-1
b
Specific Activity to gp70 SIVmac251WY30 V1V2 (Rectal secretions, week 14)
P= 0.062
6000
4000
2000
0
-2000
Vaccine
Vaccine+ IGF-1
c
Specific Activity to gp70 SIVsmE660-BR-CG7V V1V2 (Rectal secretions, week 14)
P= 0.010
10000
5000
0
-5000
Vaccine
Vaccine+ IGF-1
d
Specific Activity to gp70 SIVsmE660-BR-CG7V V1V2 (Vaginal secretions, week 14)
P= 0.021
8000
6000
4000
2000
0
-2000
Vaccine
Vaccine+ IGF-1

Figure S1

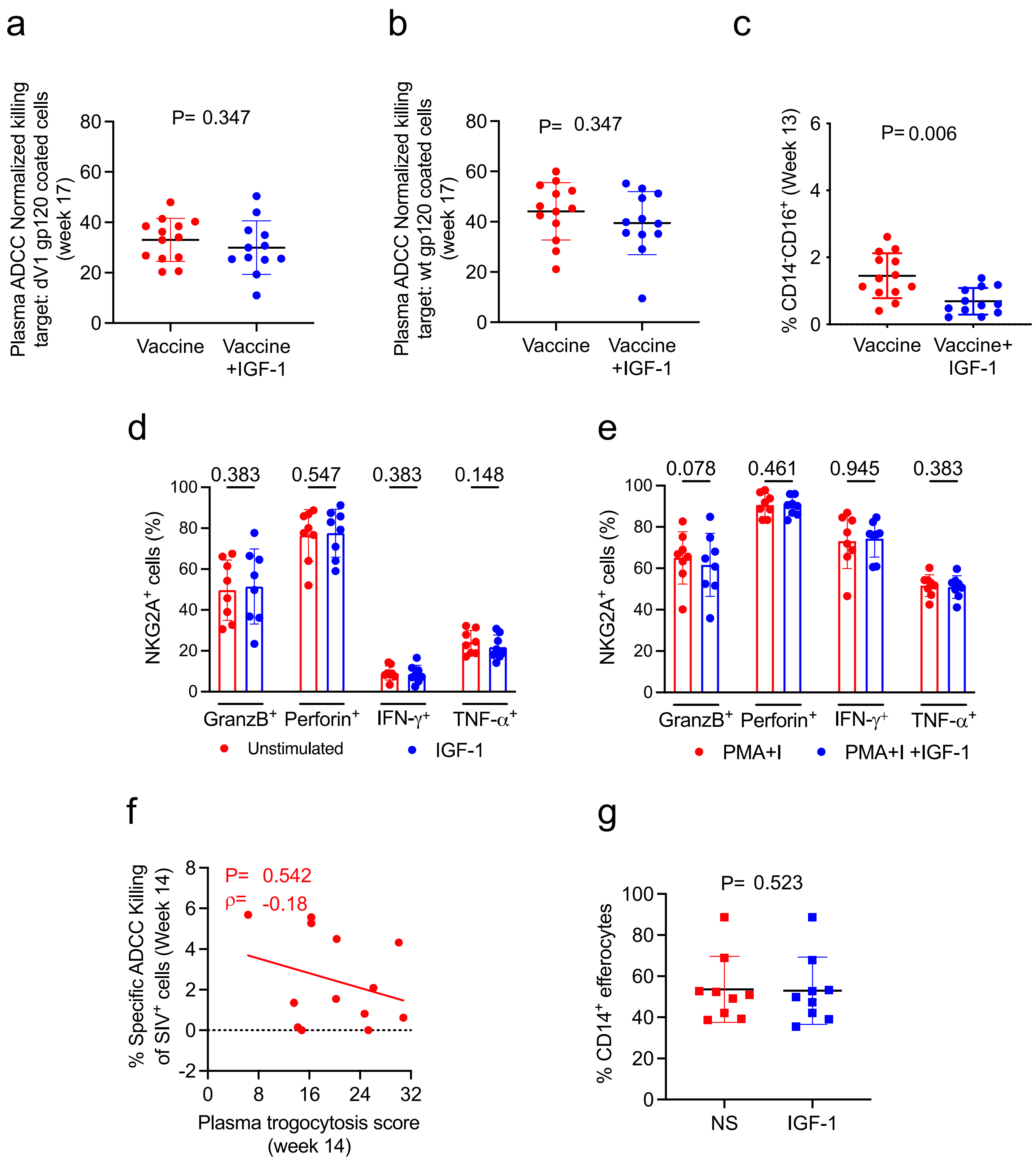
a
Plasma ADCC Normalized killing target: dV1 gp120 coated cells (week 17)
P= 0.347
Vaccine
Vaccine +IGF-1
b
Plasma ADCC Normalized killing target: wt gp120 coated cells (week 17)
P= 0.347
Vaccine
Vaccine +IGF-1
c
% CD14-CD16+ (Week 13)
P= 0.006
Vaccine
Vaccine+ IGF-1
d
NKG2A+ cells (%)
0.383
0.547
0.383
0.148
GranzB+
Perforin+
IFN-γ+
TNF-α+
Unstimulated
IGF-1
e
NKG2A+ cells (%)
0.078
0.461
0.945
0.383
GranzB+
Perforin+
IFN-γ+
TNF-α+
PMA+I
PMA+I +IGF-1
f
% Specific ADCC Killing of SIV+ cells (Week 14)
P= 0.542
ρ= -0.18
Plasma trogocytosis score (week 14)
g
% CD14+ efferocytes
P= 0.523
NS
IGF-1

Figure S2

Table S1

	Vaccine					Vaccine+IGF-1					Vaccine vs Vaccine+IGF-1
	25% Percentile	Median	75% Percentile	95% CI	N animals	25% Percentile	Median	75% Percentile	95% CI	N animals	Mann-Whitney Test P-value
**IgA**	0.82	2.62	4.8	0.75-5.86	13	0.7425	1.145	1.743	0.74-1.81	12	0.0699
**IgG1**	186.4	205.8	308.2	181.5-330.9	13	202.8	238	375.6	201.5-379	12	0.4059
**IgG2**	3.05	9.2	17.4	1.3-17.9	13	1.225	6.35	46.63	1.1-51	12	0.9245
**IgG3**	0.03	0.03	0.03	0.03-0.03	13	0.03	0.03	0.03	0.03-0.03	12	>0.9999
**IgG4**	0.04	0.04	0.065	0.04-0.07	13	0.04	0.04	0.075	0.04-0.08	12	0.8130
**Sum IgGs**	197.9	212.2	319.7	194.5-348.9	13	209.1	280.4	376.9	208.8-380.6	12	0.3980

Table S2

		Vaccine				Vaccine+IGF-1				Vaccine vs Vaccine+IGF-1
		25% Percentile	Median	75% Percentile	95% CI	25% Percentile	Median	75% Percentile	95% CI	Mann-Whitney Test P-value
Neutralization ID50	**TIER 1A SIVmac251.6 (ID#1636DB2)**	**65296**	**88146**	**166015**	**65121-171251**	**45649**	**63065**	**80256**	**43355-81232**	**0.0257**
	TIER 1A SIVsmE660/BR-CG7G.IR1 (ID#1370DB2)	5427	26438	95260	5318-116559	11495	21722	50420	11144-53496	0.8517
	TIER 1B SIVsmE660/BR-CG7V.IR1 (ID#1634DB2)	0	880	5639	0-6480	0	127.5	1017	0-1117	0.2583
	Challenge virus SIVmac251 DAY 8 2010 (ID#2079DB2)	46.5	63	112	46-119	35.5	47.5	61.25	34-63	0.1004
Neutralization ID80	TIER 1A SIVmac251.6 (ID#1636DB2)	0	1352	5673	0-5816	59.25	2398	4474	45-4503	0.4910
	TIER 1A SIVsmE660/BR-CG7G.IR1 (ID#1370DB2)	755.5	5847	22169	523-22477	4158	6128	8694	4147-9087	>0.9999
	TIER 1B SIVsmE660/BR-CG7V.IR1 (ID#1634DB2)	0	0	0	0-0	0	0	0	0-0	>0.9999
	Challenge virus SIVmac251 DAY 8 2010 (ID#2079DB2)	0	0	0	0-0	0	0	0	0-0	>0.9999

Table S3

Peptide	Amino acid sequence
15	PCVKLSPLCITMRCNKSETD
16	PLCITMRCNKSETDRWGLTK
17	RCNKSETDRWGLTKSSTTIT
18	TDRWGLTKSSTTITTAAPTS
19	TKSSTTITTAAPTSAPVSEK
20	ITTAAPTSAPVSEKIDMVNE
21	TSAPVSEKIDMVNETSSCIA
22	EKIDMVNETSSCIAQNNCTG
23	NETSSCIAQNNCTGLEQEQM
24	IAQNNCTGLEQEQMISCKFT
25	TGLEQEQMISCKFTMTGLKR
26	QMISCKFTMTGLKRDKTKEY
27	FTMTGLKRDKTKEYNETWYS
28	KRDKTKEYNETWYSTDLVCE
29	EYNETWYSTDLVCEQGNSTD

Table S4

		Vaccine					Vaccine+IGF-1					Vaccine vs Vaccine+IGF-1
		25% Percentile	Median	75% Percentile	95% CI	N animals	25% Percentile	Median	75% Percentile	95% CI	N animals	Mann-Whitney Test P-value
Plasma	MFI SIV p55	4131	14383	24772	4122-24964	12	4834	13995	27328	4085-28489	12	0.887
	MFI SIV gp41	122	167.5	452.3	113-465	10	35.5	215.5	371.3	0-376	12	0.733
	MFI SIV gp120 mac239	3807	4502	8907	3770-9334	12	2222	7821	10242	2151-10384	12	0.671
	MFI gp140 (SIVsmE660)	4407	5485	13872	4305-16191	12	6647	9980	14502	6638-14657	12	0.291
	MFI gp130 SIVmac251	5542	8859	14836	5318-16159	12	8909	15088	16134	8223-16342	12	0.198
	MFI gp70-SIVmac239cs, 23, V1V2	1186	3511	21579	830-21987	11	4879	12805	22091	3755-23352	12	0.190
	MFI gp70-SIVmac251WY30, V1V2	1223	5125	11731	687-20871	11	8697	13122	21780	8562-23437	12	0.104
	MFI gp70-SIVSME660-BR-CG7V, V1V2	1037	4631	7496	931-20893	11	5919	14453	23776	5567-24616	12	0.079
Rectal secretions	Specific activity to SIV p55	0	490	1307	0-1559	13	194.8	856.5	2347	185-2688	12	0.279
	Specific activity to SIV gp41	0	0	0	0-0	13	0	0	0	0-0	12	>0.999
	Specific activity to SIV gp120 mac239	267	845	2182	0-2423	13	579.8	1495	2125	579-2283	12	0.538
	Specific activity to gp140 (SIVsmE660)	0	710	1731	0-1792	13	91.25	917.5	1803	0-1980	12	0.766
	Specific activity to gp130 SIVmac251	1279	1696	4114	1241-4195	13	2024	3303	3974	1929-4055	12	0.650
	**Specific activity to gp70-SIVmac239cs, 23, V1V2**	**0**	**0**	**967**	**0-1459**	**13**	**322.5**	**1387**	**3147**	**307-3273**	**12**	**0.047**
	**Specific activity to gp70-SIVmac251WY30, V1V2**	**0**	**0**	**1262**	**0-1640**	**13**	**192.5**	**1660**	**2420**	**0-2450**	**12**	**0.062**
	**Specific activity to gp70-SIVsmE660-BR-CG7V, V1V2**	**0**	**0**	**411**	**0-453**	**13**	**389**	**1589**	**2976**	**189-3050**	**12**	**0.010**
Vaginal Secretions	Specific activity to SIV p55	334.8	1086	1508	148-1534	10	179.8	358	1005	122-4595	8	0.408
	Specific activity to SIV gp41	0	0	0	0-0	10	0	0	0	0-54	8	0.739
	Specific activity to SIV gp120 mac239	470.5	976.5	1544	277-2115	10	505.3	1223	2113	227-2489	8	0.762
	Specific activity to gp140 (SIVsmE660)	253	910	2395	76-2574	10	512	1148	2239	125-4706	8	0.696
	Specific activity to gp130 SIVmac251	823.8	1902	2891	394-3124	10	1153	1604	2684	0-3969	8	0.897
	Specific activity to gp70-SIVmac239cs, 23, V1V2	80.5	369.5	1433	70-3876	10	448.3	1514	3200	96-3701	8	0.203
	Specific activity to gp70-SIVmac251WY30, V1V2	50.5	590	1911	22-3502	10	943.3	1302	2397	127-3333	8	0.237
	**Specific activity to gp70-SIVsmE660-BR-CG7V, V1V2**	**90.75**	**385**	**592**	**78-817**	**10**	**1026**	**1443**	**1920**	**107-3331**	**8**	**0.021**
